# Effectiveness of Single vs Multiple Doses of Prophylactic Intravenous Antibiotics in Implant-Based Breast Reconstruction: A Randomized Clinical Trial

**DOI:** 10.1001/jamanetworkopen.2022.31583

**Published:** 2022-09-16

**Authors:** Jessica Gahm, Anna Ljung Konstantinidou, Jakob Lagergren, Kerstin Sandelin, Martin Glimåker, Hemming Johansson, Marie Wickman, Jana de Boniface, Jan Frisell

**Affiliations:** 1 Department of Reconstructive Plastic Surgery, Karolinska University Hospital, Stockholm, Sweden; 2 Department of Molecular Medicine and Surgery, Karolinska Institutet, Stockholm, Sweden; 3 Department of Surgery, Capio St Göran’s Hospital, Stockholm, Sweden; 4 Department of Breast and Endocrine Surgery, Karolinska University Hospital, Stockholm, Sweden; 5 Department of Infectious Diseases, Karolinska University Hospital, Stockholm, Sweden; 6 Unit of Infectious Diseases, Department of Medicine, Karolinska Institutet, Stockholm, Sweden; 7 Department of Oncology-Pathology, Karolinska Institutet, Stockholm, Sweden; 8 Department of Health Promotion Science, Sophiahemmet University, Stockholm, Sweden

## Abstract

**Importance:**

Multiple-dose antibiotic prophylaxis is widely used to prevent infection after implant-based breast reconstruction despite the lack of high-level evidence regarding its clinical benefit.

**Objective:**

To determine whether multiple-dose antibiotic prophylaxis is superior to single-dose antibiotic prophylaxis in preventing surgical site infection (SSI) after implant-based breast reconstruction.

**Design, Setting, and Participants:**

This prospective, multicenter, randomized clinical superiority trial was conducted at 7 hospitals (8 departments) in Sweden from April 25, 2013, to October 31, 2018. Eligible participants were women aged 18 years or older who were planned to undergo immediate or delayed implant-based breast reconstruction. Follow-up time was 12 months. Data analysis was performed from May to October 2021.

**Interventions:**

Multiple-dose intravenous antibiotic prophylaxis extending over 24 hours following surgery, compared with single-dose intravenous antibiotic. The first-choice drug was cloxacillin (2 g per dose). Clindamycin was used (600 mg per dose) for patients with penicillin allergy.

**Main Outcomes and Measures:**

The primary outcome was SSI leading to surgical removal of the implant within 6 months after surgery. Secondary outcomes were the rate of SSIs necessitating readmission and administration of intravenous antibiotics, and clinically suspected SSIs not necessitating readmission but oral antibiotics.

**Results:**

A total of 711 women were assessed for eligibility, and 698 were randomized (345 to single-dose and 353 to multiple-dose antibiotics). The median (range) age was 47 (19-78) years for those in the multiple-dose group and 46 (25-76) years for those in the single-dose group. The median (range) body mass index was 23 (18-38) for the single-dose group and 23 (17-37) for the multiple-dose group. Within 6 months of follow-up, 30 patients (4.3%) had their implant removed because of SSI. Readmission for intravenous antibiotics because of SSI occurred in 47 patients (7.0%), and 190 women (27.7%) received oral antibiotics because of clinically suspected SSI. There was no significant difference between the randomization groups for the primary outcome implant removal (odds ratio [OR], 1.26; 95% CI, 0.69-2.65; *P* = .53), or for the secondary outcomes readmission for intravenous antibiotics (OR, 1.18; 95% CI, 0.65-2.15; *P* = .58) and prescription of oral antibiotics (OR, 0.72; 95% CI, 0.51-1.02; *P* = .07). Adverse events associated with antibiotic treatment were more common in the multiple-dose group than in the single-dose group (16.4% [58 patients] vs 10.7% [37 patients]; OR, 1.64; 95% CI, 1.05-2.55; *P *= .03).

**Conclusions and Relevance:**

The findings of this randomized clinical trial suggest that multiple-dose antibiotic prophylaxis is not superior to a single-dose regimen in preventing SSI and implant removal after implant-based breast reconstruction but comes with a higher risk of adverse events associated with antibiotic treatment.

**Trial Registration:**

EudraCT 2012-004878-26

## Key Points

**Question:**

Do rates of postoperative surgical site infections after implant-based breast reconstruction improve when using a multiple-dose instead of a single-dose prophylactic antibiotic regimen?

**Findings:**

In this randomized clinical trial, there were no differences in rates of postoperative infections among patients managed with single vs multiple doses of prophylactic antibiotics; multiple doses were associated with higher rates of adverse events.

**Meaning:**

Multiple-dose intravenous antibiotic prophylaxis is not superior to a single-dose regimen in preventing postoperative infection after implant-based breast reconstruction and is not recommended because of the associated higher rates of adverse events.

## Teaser

This randomized clinical trial examines whether multiple-dose antibiotic prophylaxis is superior to single-dose antibiotic prophylaxis in preventing surgical site infection after implant-based breast reconstruction.

## Introduction

Surgical site infection (SSI), an infection that occurs after surgery in the part of the body where the surgery took place, can occur after any surgical procedure and is one of the leading causes of postoperative complications in implant-based postmastectomy breast reconstruction.^1,2,3,4,5^ Infections after implant-based reconstruction range from mild cellulitis requiring oral antibiotics to more severe cellulitis requiring intravenous antibiotic treatment. Advanced or prolonged infection can result in abscess formation and/or wound breakdown, requiring the implant to be removed because antibacterial drugs lack the ability to penetrate the bacterial film developing on the implant surface. For the affected individual, the consequences of infection after implant-based breast reconstruction can be severe; adjuvant oncological treatment may be delayed if reconstruction was attempted in the immediate setting, the implant may need to be removed despite antibiotic treatment, and in the retained implant, infection can lead to pronounced capsular contracture necessitating multiple revisional surgical procedures.^6^

Patient-related risk factors for SSI in implant-based breast reconstruction are high age, smoking, obesity, and diabetes.^7,8,9,10^ Radiotherapy increases the risk of SSI,^8,11,12,13,14^ whereas chemotherapy^12,15,16^ has not been confirmed as a risk factor for SSI in implant-based reconstruction but may increase the risk of implant loss.^16,17^

In breast implant surgery, skin-residing microorganisms, especially staphylococci, are mainly responsible for wound infection.^5,18,19,20^ Thus, best practice standards for perioperative routines including antibiotic prophylaxis are well established in implant-based breast reconstruction.^21,22,23,24,25,26^ There are several retrospective studies^20,27,28,29,30^ and reviews^4,31,32,33^ evaluating antibiotic prophylactic strategies in implant-based breast reconstruction, ranging from 1 dose preoperatively to prolonged treatment for several days or even weeks, or until any drains in the implant pocket are removed. None of these studies nor one small randomized study^34^ could show a reduction of SSI rates through prolonged prophylaxis exceeding 24 hours after implant-based breast reconstruction. Even though a single preoperative antibiotic dose offers sufficient SSI prevention in breast augmentation surgery,^35^ extended antibiotic prophylaxis is commonly given.^36,37^ It is important to consider in the context of an increased risk of acquired antibiotic resistance through extended antibiotic prophylaxis.^20^ The main aim of this prospective randomized clinical trial was to investigate whether single-dose or multiple-dose antibiotic prophylaxis is most effective in preventing implant removal and reducing SSI rates.

## Methods

### Design

**Figure 1.**
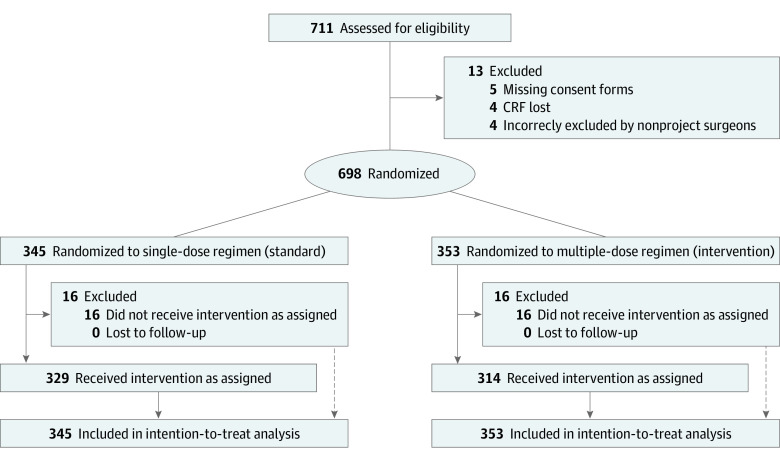
CONSORT Flow Diagram CRF indicates case report form.

This trial was conducted as a multicenter, randomized clinical superiority trial at 7 hospitals (8 departments) in Sweden from April 25, 2013, to October 31, 2018 (Figure 1). The trial protocol was approved by The Swedish Medical Products Agency and the Regional Ethical Committee. The trial protocol is available in Supplement 1. This report follows the Consolidated Standards of Reporting Trials (CONSORT) reporting guideline for randomized clinical trials.

### Trial Population

Women were eligible for inclusion if they were aged 18 years or older, willing and able to give written informed consent, and planned for immediate or delayed implant-based breast reconstruction. Women with a known allergy to both trial drugs (ie, cloxacillin and clindamycin) were not eligible. Mandatory written consent was obtained before enrollment in the trial, and enrollment was performed by surgeons.

### Randomization and Intervention

A computer-generated randomization list with permutated blocks of 50 patients was used.^38^ Participating centers were supplied with blocks consisting of sealed sequentially numbered envelopes containing information regarding the randomization result (ie, single-dose or multiple-dose antibiotic prophylaxis). Women were randomly assigned (1:1) by allocation of the next sequentially numbered envelope by a nurse or study coordinator. Neither staff nor patient was blinded to allocation.

### Surgical Technique

A nipple-sparing technique was used when the oncological and technical situation allowed it. Biological or synthetic mesh were very rarely used in Sweden during this trial’s inclusion period, and the prepectoral technique had not yet been introduced.

Implant cavity irrigation with antibiotics is not standard in Sweden in reconstructive cases; however, glove change before implant insertion is, and drains are generally used, 1 in the subpectoral and 1 in the subcutaneous position. No special support dressings are prescribed. Drains are usually left in place until fluid content is less than 30 to 50 mL, but rarely longer than 1 week.

### Study Drugs

The first-choice drug cloxacillin was given at 2 g intravenously per dose, either once (single-dose regimen) or 4 times (multiple-dose regimen) within 24 hours (ie, every 6 hours). In cases of known penicillin allergy, the second-choice drug clindamycin was administered at 600 mg intravenously per dose, either once (single-dose regimen) or 3 times (multiple-dose regimen) within 24 hours (ie, every 8 hours). All prescriptions were handled according to recommended intervals stated in the drug information from the manufacturers. The antimicrobial spectrum of cloxacillin covers *Staphylococcus* and *Streptococcus* species, whereas clindamycin covers *Staphylococcus* and *Streptococcus* species, *Haemophilus influenzae*, *Moraxella catarrhalis*, *Bacterioides* species. *Clostridium* species, *Prevotella* species, *Fusobacterium* species, *Veillonella* species, and *Chlamydia trachomatis*.

### Randomization Assignments

For group A, prophylactic antibiotics were given as single intravenous dose administrated preoperatively in the operating room before the start of surgery (standard treatment). For group B, prophylactic antibiotics were given as multiple intravenous doses within 24 hours from surgery starting with the first dose administrated preoperatively in the operating room before the start of surgery (intervention).

### Data Collection

Data on outcomes, including information on subsequent antibiotic prescription for clinically suspected and/or confirmed infection, as well as adverse events associated with antibiotic treatment, were collected from medical records and by telephone interviews of participants by a study nurse at 10 days (± 3 days), 1 month (± 7 days), 3 months (± 7 days), 6 months (± 14 days), and 12 months (± 14 days) after surgery. In this trial, SSI is defined according to the Centers for Disease Control and Prevention (CDC) definition.^23^

All trial data were collected into a case report form (CRF) and included type of implant-based reconstruction (immediate vs delayed), type of axillary surgery, laterality, use of permanent implant or tissue expander, neoadjuvant and/or adjuvant chemotherapy, radiotherapy, body weight and length, body mass index (BMI; weight in kilograms divided by height in meters squared), diabetes, smoking, immunosuppressive treatment, surgical complications (bleeding or skin necrosis), adverse events associated with antibiotic treatment (rash, loose stools, thrombophlebitis, or other), clinical signs of SSI, antibiotic prescription for clinically suspected SSI, and implant removal during follow-up. Revisional surgery for other reasons than SSI during follow-up was also recorded in the CRF. Data were monitored on site by the Clinical Trials Office at Karolinska University Hospital, Stockholm, Sweden, in accordance with Good Clinical Practice.

After 5 years, an interim analysis was performed because inclusion had been slowing down. Among more than 700 enrolled patients, no outcome difference between the 2 randomization groups was found. During the same period, the Swedish Medical Products Agency conducted an inspection at 1 of the participating sites, which had recruited 18 patients. At this inspection, protocol deviations were identified, and the study was prematurely closed by the Swedish Medical Products Agency. As a result of this inspection and the interim analysis, it was decided to close enrollment into the trial and strictly monitor all included patients on site. No additional protocol deviations were identified.

### Outcome Measures

The initially conceived primary outcome was an SSI leading to implant removal within 12 months after surgery, but the time frame was amended to 6 months during the course of the trial, the rationale being that subsequent revisional surgical procedures, such as implant exchange or capsulectomy, commonly performed 6 to 12 months after reconstruction, may substantially affect the rate of SSI, which would confound the primary end point in relation to the randomization assignment. Secondary outcomes were SSI necessitating readmission to hospital and administration of intravenous antibiotics, and clinically suspected SSI requiring the prescription of oral antibiotics within 6 months after surgery.

### Statistical Analysis

For sample size calculation, we assumed a 10% implant loss rate at 12 months after surgery in patients given single-dose antibiotic prophylaxis. To detect a 50% reduction through administration of multiple-dose antibiotic prophylaxis (ie, a 5% implant loss rate at 12 months) with a significance level (α) of 5% and a power (1 − β) of 80%, the trial needed to recruit 870 patients, 435 per randomization group.

Patients were analyzed in the randomization groups (A and B) into which they had been allocated, regardless of intervention received (intention-to-treat population). Analysis was done according to a prespecified protocol and statistical analysis plan (Supplement 1). Patient demographics and tumor characteristics at trial entry are presented for each respective randomization group. Descriptive analyses present distributions as number of cases with the respective percentages for categorical variables, and means and (SDs) or medians values with their minimum and maximum values, as appropriate, for continuous variables. Differences between the randomization groups were tested using the Wilcoxon rank-sum test for continuous variables and Fisher exact test for categorical variables.

Associations between randomization assignment and the primary and secondary outcomes were evaluated using multilevel, mixed-effects logistic regression models with center included as a random effect. Results from these models are presented as odds ratios (ORs) together with their 95% CIs. *P* values from these models refer to Wald tests. All reported *P* values are 2-sided. The cumulative proportion events are presented in graphs taking the follow-up time into account. The main statistical analyses were performed according to the intention-to-treat principle, but an additional per-protocol analysis was also performed (eTable in Supplement 2). Data analysis was performed from May to October 2021. Data analysis was performed using Stata statistical software version 16 (StataCorp).

## Results

### Patients

Between April 2013 and October 2018, 711 patients from Sweden were enrolled in the trial (Figure 1). Thirteen patients were excluded because of missing consent forms (5 patients), loss of CRF documentation during a relocation process at 1 department (4 patients), or incorrect exclusion by nonproject surgeons (4 patients).

Overall, 698 randomized patients were included in the primary analysis (345 in the single-dose group and 353 in the multiple-dose group). The baseline characteristics of the 2 randomization groups were similar (Table 1) and do not show any statistically significant differences. The median (range) age was 47 (19-78) years for those in the multiple-dose group and 46 (25-76) years for those in the single-dose group. The median (range) BMI was 23 (18-38) for the single-dose group and 23 (17-37) for the multiple-dose group. In total, 647 patients (92.7%) received antibiotic prophylaxis according to their allocated intervention, whereas in 26 cases (3.7%), the correct dose of antibiotics could not be verified in the patient records during the monitoring process because of a change of provider for digital patient records at 1 hospital. These nonverified allocations are, therefore, reported as missing data in the analysis. For 25 patients (3.6%), the received antibiotic regimen deviated from the allocated intervention because of prolonged operation time and the decision of the surgeon to administer an extra dose, or because of early discharge from the hospital, preventing the administration of multiple intravenous doses. Of the primary analysis population, 683 patients (98.0%) completed 6-month follow-up, and 666 patients (95.0%) completed 12-month follow-up.

**Table 1.** Baseline Characteristics of Patients

Characteristic	Patients, No. (%) (N = 698)	
	Single-dose antibiotics (n = 345)	Multiple-dose antibiotics (n = 353)
Participating site		
Department of Reconstructive Plastic Surgery, Karolinska University Hospital	141 (40.9)	137 (38.8)
Department of Breast Surgery, Karolinska University Hospital	81 (23.5)	87 (24.6)
South General Hospital Stockholm	51 (14.8)	61 (17.3)
Capio St Göran’s Hospital	35 (10.1)	32 (9.0)
Danderyd Hospital	12 (3.5)	12 (3.4)
Halland Hospital, Halmstad	14 (4.0)	13 (3.7)
Uppsala University Hospital	9 (2.6)	9 (2.5)
Umeå University Hospital	2 (0.6)	2 (0.6)
Age at surgery, median (range), y	46 (25-76)	47 (19-78)
Body mass index, median (range)^a^	23 (17-38)	23 (17-38)
<20	38 (11.0)	33 (9.8)
20-26	202 (58.6)	209 (59.2)
25-30	89 (25.8)	92 (26.1)
>30	16 (4.6)	19 (5.4)
Missing	0	0
Nicotine use (current smoker or moist powder tobacco user)		
No	323 (93.6)	336 (95.2)
Yes	22 (6.4)	17 (4.8)
Missing	0	0
Diabetes type 1 or 2		
No	341 (98.8)	349 (98.9)
Yes	4 (1.2)	4 (1.1)
Missing	0	0
Radiation therapy (previous or adjuvant)		
No	222 (64.3)	225 (63.7)
Yes	123 (35.7)	128 (36.3)
Missing	0	0
Indication for mastectomy		
Therapeutic	207 (60.0)	220 (62.3)
Risk-reducing	113 (32.8)	109 (30.9)
Both therapeutic and risk-reducing	25 (7.2)	24 (6.8)
Missing	0	0
Type of reconstruction		
Therapeutic mastectomy (immediate or delayed reconstruction)	207 (60.0)	220 (62.3)
Immediate reconstruction	161 (46.6)	170 (48.1)
Delayed reconstruction (previous therapeutic mastectomy)	46 (13.3)	50 (14.2)
Bilateral RRM, no cancer diagnosis	113 (32.8)	109 (30.9)
Immediate reconstruction	113 (32.8)	109 (30.9)
Delayed reconstruction	0	0
Therapeutic and contralateral RRM	25 (7.2)	24 (6.8)
Therapeutic and contralateral RRM bilateral immediate reconstruction	14 (4.1)	16 (4.5)
Delayed reconstruction following previous therapeutic, and contralateral RRM with immediate reconstruction	11 (3.2)	8 (2.3)
Missing	0	0
Bilateral reconstruction		
No	217 (62.9)	224 (63.5)
Yes	128 (37.1)	129 (36.5)
Missing	0	0
Axillary surgery		
None	190 (55.1)	193 (54.8)
Yes	155 (44.9)	159 (45.2)
Sentinel lymph node biopsy	110 (31.9)	102 (29.0)
Axillary lymph node dissection	45 (13.0)	57 (16.2)
Missing	0	1 (0.3)
Type of implant		
Permanent implant	110 (32.1)	112 (31.7)
Tissue expander	230 (67.1)	239 (67.7)
Permanent and tissue expander (bilateral case)	3 (0.9)	2 (0.6)
Missing	2 (0.6)	0
Chemotherapy		
None	239 (69.4)	235 (66.6)
Neoadjuvant	33 (9.6)	41 (11.6)
Adjuvant	59 (17.1)	64 (18.1)
Neoadjuvant and adjuvant	14 (4.1)	13 (3.7)
Missing	2 (0.6)	0
Type of antibiotic prophylaxis		
Cloxacillin	321 (93.0)	322 (91.2)
Clindamycin	22 (6.4)	31 (8.8)
Different antibiotic than study drugs	1 (0.3)	0
Missing	1 (0.3)	0
Adverse events		
No	308 (89.3)	295 (83.6)
Yes	37 (10.7)	58 (16.4)
Missing	0	0

Abbreviation: RRM, risk-reducing mastectomy.

^a^ Body mass index is calculated as weight in kilograms divided by height in meters squared.

All breast reconstructions in this trial had the implants placed submuscularly. Two patients had a comment in the CRF that acellular dermal matrix was used in the reconstruction.

During follow-up, 21 patients (3.0%) underwent unplanned surgical procedures because of surgical bleeding, tumor-involved margins, or need for additional axillary surgery. An elective revisional procedure, such as implant pocket correction, implant exchange, removal or rotation of the expander filling port, nipple reconstruction, symmetrizing surgery, or liposuction, was performed in 144 women. No implant loss was reported subsequent to these additional procedures.

### Primary Outcome

**Figure 2.**
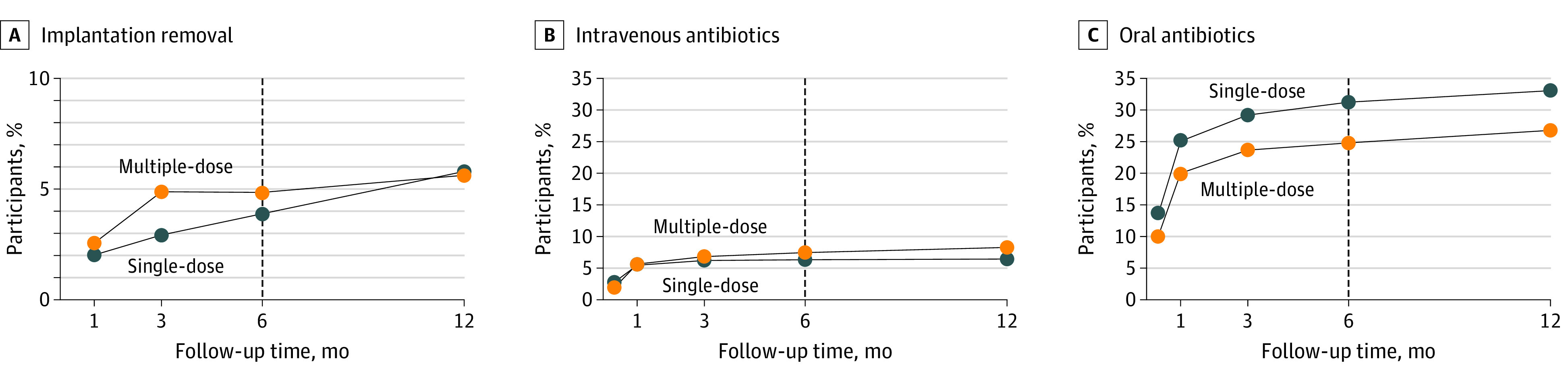
Outcomes at 6-Month Follow-up Graphs show cumulative proportion of trial participants experiencing implant removal (A), admission to hospital for intravenous antibiotics (B), and receiving prescription of oral antibiotics (C) at 6-month follow-up.

Thirty of 698 patients (4.3%) had undergone implant removal at 6-month follow-up, 13 (3.8%) in randomization group A (single-dose) and 17 (4.8%) in randomization group B (multiple-dose) (OR, 1.26; 95% CI, 0.69-2.65; *P* = .53) (Figure 2 and Table 2). There was no significant difference in time to implant removal between the 2 randomization groups (Figure 2). The implant loss rates at the different hospitals were 3.0%, 3.1%, 3.7%, 4.2,%, 4.8%, 5.6%, and 7.2%; 1 hospital had a rate of 25.0% (1 event for 4 patients recruited).

**Table 2.** Outcome at 6-Month Follow-up for Intention-to-Treat Protocol

Outcome	Patients, No. (%) (N = 698)		OR (95% CI)^a^	*P* value
	Single-dose antibiotics (n = 345)	Multiple-dose antibiotics (n = 353)		
Implant removal^b^	13 (3.8)	17 (4.8)	1.26 (0.69-2.65)	.53
Intravenous antibiotics^c^	21 (6.1)	26 (7.4)	1.18 (0.65-2.15)	.58
Oral antibiotics^d^	105 (30.4)	85 (24.1)	0.72 (0.51-1.02)	.07

^a^ Comparison is for multiple-dose vs single-dose.

^b^ Fifteen patients (2.1%) had missing outcome data (11 patients in the single-dose group and 4 patients in the multidose group).

^c^ Twenty-one patients (3.0%) had missing outcome data (16 patients in the single-dose group and 5 patients in the multidose group).

^d^ Nineteen patients (2.7%) had missing outcome data (9 patients in the single-dose group and 10 patients in the multidose group).

### Secondary Outcomes

Forty-seven patients (7.0%) had received intravenous antibiotics because of SSI at 6-month follow-up, including 21 (6.1%) in randomization group A and 26 (7.4%) in randomization group B (OR, 1.18; 95% CI, 0.65-2.15; *P* = .58) (Figure 2). There was no significant difference in time to treatment of SSI with intravenous antibiotics between the 2 randomization groups (Figure 2). The rates of intravenous antibiotic treatment for SSI at the different hospitals were 0.0%, 0.0%, 4.2%, 4.7%, 6.0%, 7.2%, and 9.0%; 1 hospital had a rate of 22.0% (4 events among 14 patients recruited).

One hundred ninety patients (27.7%) had received oral antibiotics because of a clinically suspected SSI at 6-month follow-up, including 105 patients (30.4%) in randomization group A and 85 patients (24.4%) in randomization group B (OR, 0.72; 95% CI, 0.51-1.02; *P* = .07) (Figure 2). There was no significant difference in time to treatment with oral antibiotics between the 2 randomization groups (Figure 2). The rates of oral prescriptions of antibiotics at the different hospitals were 13.3%, 15.4%, 16.7%, 21.6%, 27.8%, 33.3%, 40.0%, and 44.4%.

### Adverse Events

Adverse events likely associated with the antibiotic prophylaxis at primary intervention were reported by 95 of 698 patients (13.6%) at time of first follow-up (10 ± 3 days), including 37 (10.7%) in randomization group A and 58 (16.4%) in randomization group B (OR, 1.64; 95% CI, 1.05-2.55; *P *= .03) (Table 1). None of the adverse events was classified as serious. Loose stools were the most commonly reported adverse event and accounted for 28 of 95 events (30%), followed by rash (17 of 95 events [18%]), and thrombophlebitis (8 of 95 events [8%]).

## Discussion

This prospective, multicenter, randomized superiority clinical trial found no difference in implant loss due to SSI or postoperative antibiotic treatment for SSI between the randomization assignments single-dose vs multiple doses of intravenous antibiotic prophylaxis. These results are consistent with a recent review and meta-analysis^39^ concluding that prolonged antibiotic prophylaxis does not reduce the incidence of SSI when best practice standards for perioperative care are followed.

In the literature,^4,40^ the reported incidence of SSI after implant-based breast reconstruction ranges from 0% to 29%, with a mean of 5.8%. Because several risk factors for SSI following implant-based breast reconstruction are known, preoperative planning is key to reduce SSI rates. Prevention should begin with appropriate patient selection and choice of reconstructive timing and method. Because of the successful randomized clinical trial design, providing equal distribution of risk factors in the randomization groups, such risk factors should not affect the main results. In Sweden, a high BMI (>30) and current smoking are considered to be a relative contraindication in the guidelines^41^ for immediate breast reconstruction, which probably affected the baseline characteristics in the trial population. Patients with obesity and current smokers are more commonly planned for a delayed breast reconstruction to minimize the risk of delaying adjuvant chemotherapy and/or radiation treatment due to a postoperative complication.

The wide range of reported SSI rates may be due to divergent definitions. Some authors define SSI according to the CDC guidelines,^23^ whereas others use clinical signs of infection and subsequent outcomes such as implant removal or revisional surgery.^42^ In the present trial, we defined SSI as a confirmed or suspected infection at the surgical site, equivalent to a clinical diagnosis of infection^42^ requiring oral or intravenous administration of antibiotics. This definition may increase the number of patients receiving treatment compared with the CDC definition,^23^ but should not affect the primary outcome (ie, implant removal). Thus, the latter is probably the most reliable measure when comparing SSI rates after implant-based breast reconstruction in different reports. Because the Clavien-Dindo classification does not separate oral and intravenous antibiotic treatment, it was not applicable in this trial.^43^

The length of follow-up is an important factor associated with reported SSI rates because most cases of implant removal do not occur within the first 30 days but later during the postoperative period, sometimes as late as 1 year after surgery.^11,19,44,45,46^ Cohen et al^47^ reported the median time to implant removal to be 41 days. In their study, with a 12-month follow-up, 30 of 38 patients (80%) who lost their implant because of SSI did so within the first 6 months following surgery.^47^ Every revision surgery following implant-based breast reconstruction approximately involves a 4.7% risk of implant failure,^48^ which is associated with a history of infection after the primary intervention, diabetes, previous axillary clearance, smoking, and postmastectomy radiotherapy. In the present trial, however, 165 revisional surgical procedures were performed without leading to implant removal.

Antibiotic consumption is associated with the development of antibiotic resistance^49^ and results in additional costs and adverse events,^20,50^ such as *Clostridium difficile* infection.^51,52^ In the present trial, a larger proportion of patients allocated to multiple-dose antibiotic prophylaxis reported adverse events associated with their antibiotic treatment. An increasing variety of disorders are correlated with the host microbiota.^53^ The reported adverse events in the present trial were mainly gastrointestinal, which should underline the need to reduce the duration of prophylactic antibiotic administration to limit a potential impact on gastrointestinal health that may be long lasting.^54,55,56^ Apart from the negative impact on the individual level, the frequent overuse of antibiotic drugs in implant-based breast reconstruction counteracts efforts and strategies to combat antibiotic resistance, as outlined in The Global Action Plan on Antimicrobial Resistance adopted by the World Health Organization member states.^57^

### Limitations

This study has limitations that should be addressed. The current trial was initiated before the use of biological (acellular dermal matrix) or synthetic (absorbable and nonabsorbable) meshes and before the prepectoral implant positioning became popular.^58,59^ Thus, these techniques cannot be assessed in this trial, and the results’ applicability to such techniques is uncertain. According to recent studies,^60,61,62,63,64,65,66^ however, the prepectoral position (which, in principle, always includes some type of mesh) renders no higher SSI rates compared with the subpectoral position, so multiple-dose antibiotic prophylaxis is probably not indicated.

With the original trial design (ie, to detect an improvement from 10% to 5% in the primary outcome measure, implant removal), with standard requirements for significance (5%) and power (80%), a total of 870 patients were needed. It was deemed practically feasible to recruit this number of patients from hospitals in Sweden within a reasonable time frame; still, enrollment was slow, and the trial was closed before full inclusion. To detect an improvement of 25% (ie, from 10% to 7.5%), the trial would have needed to recruit 4000 patients, which would have been rather impossible to achieve. Since the original accrual target was not achieved, the statistical power of the trial is negatively affected, and small differences between the randomization groups cannot be ruled out.

## Conclusions

Multiple-dose antibiotic prophylaxis is not superior to a single-dose regimen in preventing implant removal due to SSI in implant-based breast reconstruction but is associated with more adverse events. Thus, multiple-dose antibiotic prophylaxis is not recommended.

## Notes

Supplement 1.
Trial Protocol and Statistical Analysis Plan

Supplement 2. **eTable.** Outcome at 6-Month Follow-up for Per-Protocol Analysis, According to Randomization (n = 647)

Supplement 3.
Data Sharing Statement
