# Supplementary material for: Effectiveness of Single vs Multiple Doses of Prophylactic Intravenous Antibiotics in Implant-Based Breast Reconstruction: A Randomized Clinical Trial
Source: JAMA Netw Open. 2022 Sep 16;5(9):e2231583. doi: 10.1001/jamanetworkopen.2022.31583 (PMC9482055; doi:10.1001/jamanetworkopen.2022.31583)
Supplement: Supplement 1. — Trial Protocol and Statistical Analysis Plan [file jamanetwopen-e2231583-s001.pdf]

Trial Protocol and Statistical Analysis Plan

The Use of Prophylactic Antibiotics in Implant-Based Immediate and Delayed Breast Reconstruction - A Prospective Randomized Trial.

Eudra CT-number: 2012-004878-26
Sponsor's protocol code number: 26842468452

Sponsor: Jakob Lagergren, M.D., senior consultant/Ph.D., Department of Reconstructive Plastic Surgery, Karolinska University Hospital, Solna, 171 76 Stockholm. +468-517 796 59, +4676-117 02 82.

The Swedish Medical Products Agency approved testing with clinical drugs.

The Research Ethics Committee in Stockholm:

Registration number: 2012/1032-31/1, update 2014/1158-32.

Date of approval: 2012-07-11, update 2014-07-04.

Research scientists

Jakob Lagergren, M.D./Senior Consultant/Ph:D., Department of Reconstructive Plastic Surgery, Karolinska University Hospital Solna, 171 76 Stockholm. +468-517 796 59, 4676-117 02 82.

Anna Ljung Konstantinidou, M.D./Consultant, Department of Breast and Endocrine Surgery, Karolinska University Hospital Solna, 171 76 Stockholm, +468-517 700 00, +4670-753 32 20.

Kerstin Sandelin, M.D./Senior Consultant/Associate Professor, Department of Breast and Endocrine Surgery, Karolinska University Hospital Solna, 171 76 Stockholm. +468-517 718 76.

Jessica Gahm, M.D./ Consultant /Ph.D., Department of Reconstructive Plastic Surgery, Karolinska University Hospital Solna, 171 76 Stockholm. +468-517 741 59.

Martin Glimåker, M.D./Senior Consultant/Ph.D., Department of Infectious Diseases, Karolinska University Hospital, 171 76 Stockholm. +468-517 791 79.

Synopsis

EudraCT-number: 2012-004878-26

Sponsor's protocol number: 26842468452

Sponsor: Jakob Lagergren, M.D./Senior Consultant/Ph:D., Department of Reconstructive Plastic Surgery, Karolinska University Hospital Solna, 171 76 Stockholm. +468-517 796 59, +4676-117 02 82.

Title: The Use of Prophylactic Antibiotics in Implant-Based Immediate and Delayed Breast Reconstruction - A Prospective Randomized TrialTrial.

Background: Breast cancer is the most common form of cancer among women in industrialized countries and surgery is the primary treatment. Today, almost all breast cancer patients undergo surgery; nearly half of the patients undergo breast-conserving surgery and the remainder undergo mastectomy.

There are different methods of breast reconstruction after mastectomy. Reconstruction may be achieved with an implant (a prosthetic filled with silicon gel or saline) or autologous, the transfer of one's own body fat from, for example, the back or abdomen that is injected into the breast. Occasionally the methods are combined. Breast reconstruction may be performed directly after a mastectomy and is then referred to as immediate reconstruction, or as a second surgery at a later date referred to as delayed reconstruction. Prophylactic antibiotics are administered in connection to certain surgical procedures to help reduce the risk of a postoperative infection. There is scientific evidence showing that prophylactic antibiotics reduce the number of infections after, for example, colorectal surgery, vascular surgery, heart surgery, pacemaker implantation, bone fracture surgery, joint prosthetics and breast cancer surgery. There are no equivalent studies for the benefits of prophylactic antibiotics in connection with reconstructive breast surgery with (an) implant. A single dose of antibiotics can, if given in connection with most surgeries, be just as effective as several doses. In connection with other types of surgeries, for example orthopedic and heart surgery, administering several doses of intravenous prophylactic antibiotics is regular praxis. The effect of the number of prophylactic doses during reconstructive breast surgery with (an) implant is not evident. Here at Karolinska University Hospital Solna we currently administer perioperative prophylactic antibiotics during reconstructive breast surgery with (an) implant as a single dose of intravenous Cloxacillin.

Objective: The objective of this trial is to ascertain which regime with prophylactic antibiotics, single dose or multiple doses, is most effective with the intention of preventing complications due to infection and side effects in connection with reconstructive breast surgery with (an) implant. We would like to optimize the use of prophylactics and in this way reduce the number of postoperative infections, minimize side effects and the overall consumption of antibiotics.

Method: This trial is carried out as a national multicenter trial including five clinics for breast and plastic surgery in Stockholm, as well as a number of clinics for breast and plastic surgery throughout the rest of the country that also perform reconstructive breast surgery with (an) implant.

Trial population: All women (over 18 years of age) planned to undergo immediate or delayed first-time reconstruction with an implant are offered participation in this trial. Exclusion criteria include the lack of being able to make a decision concerning oral and written information about the trial or an allergy to both trial drugs (Cloxacillin and Clindamycin).

Trial Arms: Participants are randomized to either of the two trial arms: A, prophylactic antibiotics given intravenously as a single preoperative dose at start of surgery. B, prophylactic antibiotics administered in multiple perioperative, intravenous doses on the day of surgery beginning preoperatively in connection to start of surgery. According to completed power calculations, at least 870 patients need to be included trial with at least 435 patients in each trial arm. Inclusion time is estimated to 3-4 years.

Randomization: Randomizing occurs according to the computer generated randomization list with block randomization (18 blocks with 50 patients per block). Numbered and sealed envelopes are then handed out to the participants in accordance with the randomization list.

Clinical trial drugs: First hand antibiotics are Cloxacillin and second hand antibiotics (in the case of penicillin allergy) are Clindamycin. In trial arm A, Cloxacillin is given in a dose of 2 g x 1 intravenously or Clindamycin in a dose of 600 mg x 1 intravenously. In trial arm B Cloxacillin is given in a dose of 2 g x 4 intravenously or Clindamycin in a dose of 600 mg x 3 intravenously, evenly spread out over 24 hours.

Outcome Measurements: This trial's outcome measurement is a postoperative infection in the reconstructed breast. In this trial, a postoperative infection is defined as the state/status of the patient that would incite the treating physician to prescribe treatment with antibiotics. The three degrees of infection that are registered are: Infection leading to the removal of an implant (primary endpoint), infection requiring intravenous treatment with antibiotics in hospital (secondary endpoint) and infection requiring treatment with oral antibiotics (secondary endpoint).

Follow-up: Follow-up is carried out 10 days (+/- 3 days), 1 month (+/- 7 days), 3 months (+/-7 days), 6 months (+/- 14 days) and 12 months (+/-14 days) after surgery. These follow-ups are performed by a clinical trials-nurse who collects patient information via patient records and interviews over the telephone.

Handling of data and patient confidentiality: Patient information will be collected in a CRF (Case Report Form) containing patient information such as which type of breast surgery the patient has undergone, if the patient has received chemotherapy and/or radiotherapy, weight, height and BMI as well as information on side effects, postoperative infections, extra doctor's visits or sick leave due to infection. All patient

information from this trial will be saved in a data base and handled confidentially. Analyses and results reports will be processed anonymously in groups. Regular patient journals will be handled confidentially in accordance with The Swedish Patient Data Act.

Regulatory Issues: Monitor will perform quality controls of the trial according to the stipulations for confidentiality. The trial will be otherwise carried out in accordance with trial protocol and GCP (Good Clinical Practice), as well as follow the current law and regulations that apply. The risk for SUSARs in this trial is minimal.

Background

Breast cancer

Breast cancer is the most common form of cancer among women in industrialized countries and accounts for up to 25% of all cancer types. Approximately 1 in 10 women will be affected at some point in their lives and in Sweden approximately 7000 women are affected each year (1). Surgery is the primary treatment for breast cancer and almost all women with breast cancer undergo surgery and nearly half of those patients will undergo breast-conserving surgery (2). Mastectomy is performed if the tumor is large, is multifocal or inflammatory, in the case of recurrence after earlier breast conserving surgery or after prophylactic breast surgery as may be the case in hereditary breast cancer (3). Approximately 3000 women undergo mastectomy each year in Sweden.

Breast Reconstruction

There are different methods for reconstructive breast surgery after mastectomy. Reconstruction may be achieved with implants, that is to say a prosthetic filled with silicone gel or saline, which is placed under muscle tissue in order to recreate volume and form. Autologous reconstruction is another option in which one's own body fat from, for example, the back or abdomen is transitioned to the breast. Occasionally the methods are combined. Reconstructive breast surgery may be immediate, meaning in direct connection to mastectomy, or during a later, second surgery known as delayed reconstruction (1). According to a national questionnaire for women who have undergone surgery for breast cancer between 1998 and 2003, approximately 20% of those who underwent mastectomy also received reconstruction and 1/4 of those with immediate reconstruction. Frequency of breast reconstruction varied, however, between the different regions (3). Immediate reconstruction is most common in the regions of Stockholm and Gothenburg where the rate is 19.7% (2009), while the average for Sweden is 6.1% (2009). Currently there is no data for the number of women who received delayed reconstruction (2). Karolinska University Hospital performs the most reconstructive breast surgeries with (an) implant in Sweden and we are known for our lengthy, well-documented experience and strong tradition of collaboration between breast and plastic surgeons.

Prophylactic Antibiotics and Infections

Prophylactic antibiotics are administered in connection to certain surgical procedures with the intention of reducing the risk of a postoperative infection. Infections cause the patient discomfort or suffering and are costly for the health care system and the community (4). An infection after reconstructive breast surgery with (an) implant may incite a long period with infectious symptoms (localized pain, redness, swollen breast and fever), health status deterioration and lengthy treatments with antibiotics. On occasion, an infection cannot be remedied with antibiotics and the implant must be removed and a period of 6 months should pass, without an implant, before the patient may undergo new reconstructive surgery. An infection can thus bring about great physical and mental distress for the patient, often leading to long-term sick leave. Incidence of postoperative infections in connection with reconstructive breast surgery with (an) implant varies according to the different materials used. Several different studies report numbers of 6-29% (5, 6, 7, 8, 9). In a national trial for prophylactic mastectomy among Swedish women who underwent surgery between 1995 and 2005, removal of prosthetic due to infection affected 10% of the patients (10). The medicinal advantage of prophylactic antibiotics must be weighed again the risk for side effects of prophylactics and developing antibiotic resistant bacteria. Antibiotic resistance is propelled by the total consumption of antibiotics in the community and prophylactic antibiotics may, when used correctly, reduce the overall necessitation of antibiotics (4).

There is scientific evidence showing that prophylactic antibiotics reduce the number of infections after, for example, colorectal surgery, vascular surgery, heart surgery, pacemaker implantation, bone fracture surgery, joint prosthetic surgery and breast cancer surgery. Similar studies are lacking however, as to the value of prophylactic antibiotics in connection to reconstructive breast surgery with (an) implant. Staphylococcus aureus is the major pathogen in a postoperative wound and prosthetic infections and should thereby, based upon empirical evidence, be susceptible to prophylactics. A single dose of antibiotics can for many surgeries be just as effective as several doses. In connection wit other prosthetic surgeries, such as in orthopedic and heart surgery, multiple doses of intravenous antibiotics is regular praxis. The effect of the number of prophylactic doses in connection with reconstructive breast surgery with (an) implant is not clear (4). In an American retrospective trial of different antibiotic regimes for breast cancer surgery, including immediate reconstruction with (an) implant, any difference in frequency of infection could not be proven between patients who received preoperative prophylactic antibiotics and those who received both pre- and postoperative prophylactic antibiotics (11). In yet another American retrospective trial however, in the case of delayed reconstruction with (an) implant among women who have undergone radiotherapy, the result was prolonged prophylactic antibiotics with less frequent postoperative infections (12).

Objectives and Questions

Where breast cancer surgery is concerned there is scientific evidence that a single dose of antibiotics as prophylaxis reduces the risk of postoperative infections. As far as evidence for prophylactic antibiotics within reconstructive breast surgery, the data is minimal, therein, need for such a trial concerning this matter. In an investigation of existing literature on this topic performed by The Swedish Agency for Health Technology Assessment, not a single study could be found specifically for prophylactic antibiotics in connection with reconstructive breast surgery with (an) implant (4).

Currently at Karolinska University Hospital, Solna, prophylactic antibiotics are administered preoperatively as single dose of intravenous Cloxacillin in connection with reconstructive breast surgery with (an) implant. The objective of this trial is to ascertain which regime with intravenous prophylactic antibiotics, single dose or multiple doses, is the most effective as far as complications due to infection or complications with breast reconstruction with (an) implant. Our aim is to optimize prophylaxis and in this way reduce the number of postoperative infections, minimize side effects and the overSall consumption of antibiotics.

The questions are:

1. Can changing the current regime of prophylactic antibiotics reduce the number of postoperative infections in breast reconstruction with (an) implant?

2. What is the importance of the length of prophylactic treatment?

Method

Participant Sites

This trial is carried out as a national multicenter trial including five breast and plastic surgery hospitals and departments in Stockholm as well as a number of breast and plastic surgery clinics in the rest of the country that perform implant-based breast reconstruction with (an) implant. For contact information of participating clinics, see attachment 1. At the five participating clinics in Stockholm approximately 300 immediate and delayed implant-based reconstructive breast surgeries are performed each year.

Trial Population

Inclusion Criteria

All women (older than 18 years of age) planned to undergo immediate or delayed implant-based first-time reconstruction are asked about participation in this trial. After receiving oral and written information concerning this trial, those women wishing to participate give their written approval in a form of consent.

Exclusion Criteria

1. The inability to make a decision based upon the oral and written information about this trial.
2. Allergy to both clinical testing drugs (Cloxacillin and Clindamycin)

Trial Arms

The participants are randomized to either of the two trial arms:

A. Prophylactic antibiotics administered preoperatively as a single intravenous dose in connection with start of surgery.
B. Prophylactic antibiotics administered in multiple doses during the day of surgery starting preoperatively in connection with start of surgery.

Randomization

Randomizing occurs according to the computer-generated randomization list (www.randomization.com) with block randomization (18 blocks with 50 patients per block). Numbered and sealed envelopes that are archived in boxes then handed out to the participating sites. When a patient is chosen for inclusion, a new envelope is opened containing information about which of the two trial arms the patient is included in. The trial is unblinded for both the patients and the personnel responsible for treatment.

According to completed power calculations, at least 870 patients are included in this trial with approximately 435 patients in each trial arm. Inclusion time is calculated to 3-4 years. It is currently difficult to calculate how many patients will be included from the respective sites.

Clinical Trial Drugs

The clinical trial drugs that are used are antibiotics. First choice antibiotic is Cloxacillin and second choice antibiotic (in the case of penicillin allergy) is Clindamycin. In trial arm A, Cloxacillin is given in a dose of 2 g x 1, intravenously, or Clindamycin in a dose of 600 mg x 1, intravenously. In trial arm B Cloxacillin is given in a dose of 2 g x 4, intravenously, or Clindamycin in a dose of 600 mg x 3, intravenously, evenly spread out over 24 hours.

Surgery

Surgery is performed under general anesthesia. The implant is either an expander implant that, after surgery, is gradually filled with saline or is a pre-filled/pre-formed, permanent implant. All implants have a textured outer layer and an outer capsule of silicon. The implant will be handled in a standardized manner according to mutual instructions at participating clinics.

Outcome Measurements

This trial's outcome measurement is a postoperative infection in the reconstructed breast. In this trial, a postoperative infection is defined as the state/status of the patient that will incite the treating physician to prescribe treatment with antibiotics. The three degrees of infection that are registered are:

1. Infection leading to the removal of an implant (primary endpoint).

2. Infection requiring intravenous treatment with antibiotics in hospital (secondary endpoint).

3. Infection requiring treatment with oral antibiotics (secondary endpoint).

Furthermore, side effects caused by given prophylactic antibiotics are registered.

Follow-ups

Follow-up of patients is carried out 10 days (+/- 3 days), 1 month (+/- 7 days), 3 months (+/-7 days), 6 months (+/- 14 days) and 12 months (+/-14 days) after surgery. These follow-ups are performed by a clinical trials-nurse who collects patient information via patient records and interviews over the telephone.

Handling of Data and Patient Confidentiality

Patient information will be collected in a CRF (Case Report Form) that will be stored and locked and only a clinical research nurse will have access to them. The CRF contains patient information such as which type of breast surgery the patient has undergone, if the patient has received chemotherapy and/or radiotherapy, weight, height and BMI as well as information on side effects, postoperative infections, extra doctor's visits or sick leave due to infection. All patient information from this trial will be saved in a registry/database and handled confidentially, only a clinical research nurse and those technicians involved in this trial will have access to this data. All collected data will be saved for five years after the trial is published. Analyses and results reports will be processed anonymously in groups. Regular patient journals will be handled confidentially in accordance with The Swedish Patient Data Act.

The patient retains the right to, at any time and without further explanation, terminate participation in the trial. If so requested, all trial-related documents and information will be permanently deleted or destroyed. Should the patient choose to terminate participation in this trial, the time and reason, if one is given, for termination will be registered in the CRF and later reported with the results of the trial.

Regulatory Issues

Monitor will perform quality controls of the trial according to the stipulations for confidentiality. The trial will be otherwise carried out in accordance with trial protocol and GCP (Good Clinical Practice), as well as follow the current law and regulations that apply. If any changes should occur in the trial plan it will be added into the trial protocol (as an update in the version number) and reported to The Swedish Medical Products Agency.

Eventual serious incidents are reported through direct contact between the clinical trial investigator and sponsor where each trial unit/hospital is appointed a research nurse who will file the report. These potential incidents will be documented in the database. Furthermore, regular controls of the participants at the follow-ups as well as a review of the participant's journals where eventual incidents are reported to the sponsor by the

research nurse responsible for these follow-ups. The risk for SUSARs in this trial is minimal but should it occur, will be reported in EMA's database (London) in a specific form provided by KTA (Karolinska Trial Alliance).

Financing

This trial will mainly be carried out within the framework of clinical work done at the participating clinics. For costs outside of these, financing will be provided by ALF Medicine, project funding via the Clinic for Reconstructive Plastic Surgery, Karolinska University Hospital, Solna (ALF-representative Marie Wickman-Chantereau, M.D./Division Manager/Professor, +468 517 700 00.

Statistics

The difference in the proportion of patients in the comparison groups who develop infections or don't, will be counted, as well as will the equivalency of the 2-sided, 95% confidence interval. This trial analyses in the same manner as a superiority trial where trial medications are expected to be superior to the comparison group's medication with the occurrence of infection as the focus. The difference between the two comparison groups will be analyzed with a T-test or a Mann-Whitney-test. The Chi2-test will be used to calculate categorical variables as long as the number of patients in each cell is at least five. In the case of fewer patients, Fisher's exact test will be used. Our primary analysis strategy is "intention to treat", that is to say all patients randomized to their respective intervention will be included in the group they were randomized to no matter which treatment they later receive. One per-protocol analysis will also be performed as a sensitivity test, that is to say, the result will be analyzed according to which treatment the patients actually received without regard to randomization. This is however a secondary test compared to the "intention to treat". In the case of dropout after randomization, the patients will be included in the group they were initially randomized to.

Publication

Trial results will be presented via one or several articles published in appropriate peer-reviewed scientific journals. Keeping in mind the number of patients that will be included and the follow-up time, this publication will most likely take place about five years after initiation of the trial. The article or articles will even be included in postgraduate doctoral thesis/dissertation.

Ethical Questions

In this trial there is no placebo control group. The thought of including a group of patients without prophylactic antibiotics at the time of implantation of, for example a pacemaker, which is a type of surgery known as having an equally prevalent risk for infection as reconstructive breast surgery with (an) implant and where previous studies

have shown that prophylactic antibiotics are of significant value, can be seen as unethical.

Other ethical questions that arise are:

- If daily prophylactic antibiotics are more effective than a preoperative dose to avoid complications from infection, are we subjecting those patients who receive single dose prophylaxis to an increased risk of infection?

- Are we subjecting patients who receive multiple doses of prophylaxis for unnecessary amounts of antibiotics and with increased side effects as a result?

Significance

As of today, the scientific evidence is insufficient for assessing the value of prophylactic antibiotics in connection with reconstructive breast surgery with (an) implant, which is why there is a need for a trial to increase our knowledge on this subject. Should we, through this trial, succeed in deducing which prophylactic antibiotic regime is the most effective as far as risk for infection and side effects in connection with reconstructive breast surgery with (an) implant are concerned, then the risk for postoperative infections and re-operations could be reduced and therein even the patient's suffering and discomfort would diminish. A prospective, randomized trial in this regard would therefore fulfill an important function in the field of reconstructive breast surgery.

References

Date and location: ….

Sponsor's signature: ….

Sponsor's name printed: …..
