# Supplementary material for: Effectiveness of Single vs Multiple Doses of Prophylactic Intravenous Antibiotics in Implant-Based Breast Reconstruction: A Randomized Clinical Trial
Source: JAMA Netw Open. 2022 Sep 16;5(9):e2231583. doi: 10.1001/jamanetworkopen.2022.31583 (PMC9482055; doi:10.1001/jamanetworkopen.2022.31583)
Supplement: Supplement 2. — eTable. Outcome at 6-Month Follow-up for Per-Protocol Analysis, According to Randomization (n = 647) [file jamanetwopen-e2231583-s002.pdf]

Supplemental Online Content

Gahm J, Ljung Konstantinidou A, Lagergren J, et al. Effectiveness of single vs multiple doses of prophylactic intravenous antibiotics in implant-based breast reconstruction: a randomized clincial trial. *JAMA Netw Open*. 2022;5(9):e2231583.
doi:10.1001/jamanetworkopen.2022.31583

eTable. Outcome at 6-Month Follow-up for Per-Protocol Analysis, According to Randomization (n = 647)

This supplemental material has been provided by the authors to give readers additional information about their work.

© 2022 Gahm J et al. *JAMA Network Open.*

eTable. Outcome at 6-Month Follow-up for Per-Protocol Analysis, According to Randomization (n = 647)

Outcome	Single-dose (%) N=321	Multiple-dose (%) N=326	Odds-ratio[a] (95% confidence interval)	p-value
Implant removal[b]	12 (3.7)	16 (4.9)	1.31 (0.61-2.82)	0.487
Intravenous antibiotics[c]	18 (5.6)	24 (7.4)	1.30 (0.69-2.45)	0.413
Oral antibiotics[d]	97 (30.2)	77 (23.6)	0.71 (0.50-1.02)	0.063

[a]Multiple-dose versus single-dose.
[b]12 patients (1.9%) with missing outcome data (8/4).
[c]18 patients (2.8%) with missing outcome data (13/5).
[d]18 patients (2.8%) with missing outcome data (8/10).

© 2022 Gahm J et al. *JAMA Network Open.*
