# Supplementary material for: Effectiveness of Single vs Multiple Doses of Prophylactic Intravenous Antibiotics in Implant-Based Breast Reconstruction: A Randomized Clinical Trial
Source: JAMA Netw Open. 2022 Sep 16;5(9):e2231583. doi: 10.1001/jamanetworkopen.2022.31583 (PMC9482055; doi:10.1001/jamanetworkopen.2022.31583)
Supplement: Supplement 3. — Data Sharing Statement [file jamanetwopen-e2231583-s003.pdf]

# Data Sharing Statement

Gahm. Effectiveness of Single vs Multiple Doses of Prophylactic Intravenous Antibiotics in Implant-Based Breast Reconstruction. *JAMA Netw Open*. Published September 16, 2022. doi:10.1001/jamanetworkopen.2022.31583

**Data**
**Data available:** Yes
**Data types:** Deidentified participant data
**How to access data:** data can be made available from the authors upon reasonable request for meta-analyses, and with the appropriate approvals of the Swedish Review Authority. Data sharing is possible beginning three months and ending five years following article publication. Proposals should be directed to the corresponding author; to gain access, data requestors will need to sign a data access agreement. Jessica.gahm@regionstockholm.se
**When available:** With publication

**Supporting Documents**
**Document types:** None

**Additional Information**
**Who can access the data:** data can be made available from the authors upon reasonable request for meta-analyses, and with the appropriate approvals of the Swedish Review Authority. Data sharing is possible beginning three months and ending five years following article publication. Proposals should be directed to the corresponding author; to gain access, data requestors will need to sign a data access agreement.
**Types of analyses:** data can be made available from the authors upon reasonable request for meta-analyses, and with the appropriate approvals of the Swedish Review Authority. Data sharing is possible beginning three months and ending five years following article publication. Proposals should be directed to the corresponding author; to gain access, data requestors will need to sign a data access agreement.
**Mechanisms of data availability:** data can be made available from the authors upon reasonable request for meta-analyses, and with the appropriate approvals of the Swedish Review Authority. Data sharing is possible beginning three months and ending five years following article publication. Proposals should be directed to the corresponding author; to gain access, data requestors will need to sign a data access agreement.
